# Supplementary material for: Impact of universal varicella vaccination on the use and cost of antibiotics and antivirals for varicella management in the United States
Source: PLoS One. 2022 Jun 10;17(6):e0269916. doi: 10.1371/journal.pone.0269916 (PMC9187103; doi:10.1371/journal.pone.0269916)
Supplement: S1 Appendix — (DOCX) [file pone.0269916.s001.docx]

S1 Appendix

Contents

[Parameters 2](#_Toc102573831)

- [Table A. Model inputs for varicella cases by vaccination status for a cross-section of 2017 cohort 2](#_Toc102573832)
- [Table B. Treatment parameters by complication status 2](#_Toc102573833)

[Prescription Cost Calculations 2](#_Toc102573834)

- [Table C. Average antiviral treatment cost 3](#_Toc102573835)
- [Table D. Average antibiotic treatment cost 3](#_Toc102573836)

[Vignettes used for Calculations 5](#_Toc102573837)

- [Table E. Vignettes used for analysis 5](#_Toc102573838)

[Modeling results 6](#_Toc102573839)

- [Table F. Base case scenario: Annual varicella prescriptions and costs for total, varicella cases with and without complications 6](#_Toc102573840)
- [Table G. Annual prescription cost for base case scenarios 7](#_Toc102573841)
- [Table H. Annual varicella prescriptions for scenarios with varying vaccination coverage (Base case, 0%, 20%, 80% vaccination coverage scenarios) 7](#_Toc102573842)
- [Table I. Annual varicella prescription costs for scenarios with varying vaccination coverage (Base case, 0%, 20%, 80% vaccination coverage scenarios) 9](#_Toc102573843)
- [Table J. Data for Figure 2: Annual prescriptions for varicella cases with no complications 10](#_Toc102573844)
- [Table K. Data for Figure 3: Annual prescriptions for varicella cases with complications 10](#_Toc102573845)
- [Table L. Data for Figure 4: Annual varicella prescription costs 10](#_Toc102573846)

[References 11](#_Toc102573847)

## Parameters

### Table A. Model inputs for varicella cases by vaccination status for a cross-section of 2017 cohort

| Row | Definition | Unvaccinated | 1-Dose Vaccinated | 2-Dose Vaccinated | Total | Source |
| --- | --- | --- | --- | --- | --- | --- |
| #1 | Proportion of children <18 years of age in 2017 by vaccination status | 3.8% | 2.4% | 93.8% | 100% | Mellerson 2018[1],  See footnote ^a^ |
| #2 | Population distribution of children <18 years of age in 2017 by vaccination status | 2,796,155 | 1,765,992 | 69,020,868 | 73,583,015 | U.S. Census Bureau, 2017;  See footnote ^b^ |
| #3 | Average annual incidence rate by vaccination status | 14.0% | 0.8% | 0.2% | NA | Kuter 2004[2] |
| #4 | Number of varicella cases in 2017 by vaccination status | 396,633 | 14,406 | 140,395 | 551,434 | See footnote ^c^ |
| #5 | Average annual rate of varicella complications | 6.7% | 2.5% | 2.0% | NA | Derived from Kuter 2004[2] |
| #6 | Annual number of varicella cases with complications | 27,007 | 378 | 2,906 | 30,291 | See footnote ^d^ |
| #7 | Annual number of varicella cases with no complications | 369,625 | 14,029 | 137,490 | 521,143 | See footnote ^e^ |

* Numbers in this table are rounded up.

Calculations

^a) From Table 1 of Mellerson at al 2018 for the 2017-2018 school year, the median 2-dose varicella vaccination coverage for the country was 93.8% ; while that for 1 dose varicella vaccination was 96.2%.[1] This was used to calculate proportion of children of <18 year olds in 2017 vaccinated with a single dose of varicella vaccine (96.2% - 93.8% = 2.4%); and the proportion who were unvaccinated (remaining = 100% - 93.8% - 2.4% = 3.8%) in Row#1^

^b) Population per US Census of individuals <18 years of age in 2017=73,583,015. This number was multiplied by 3.8%, 2.4% and 93.8% respectively to get the population by vaccination status in 2017 among <18 year olds in the US in Row#2^

^c) To calculate the number of varicella cases in 2017 by vaccination status in Row#4, the populations in Row #2 for unvaccinated, 1 dose and 2 dose groups were multiplied by their respective average annual incidence rates in Row#3. Summing the number of varicella cases across the 3 groups = 551,434 total varicella cases in 2017 among children <18 years in 2017^

^d) Derived from row #4 and #5 for each vaccination group and then summed up.^

^e) To calculate the number of varicella cases with no complications, subtract Row #6 from Row#4^

### Table B. Treatment parameters by complication status

| Treatment Parameters | Varicella with no complications | Varicella with complications | Source |
| --- | --- | --- | --- |
| Proportion prescribed antivirals | 15.9% | 14.1% | Vignettes survey[3] |
| Proportion prescribed antibiotics | 5.0% | 41.5% | Vignettes survey[3] |
| Proportion prescribed both antibiotics and antiviral | 0.4% | 8.5% | Vignettes survey[3] |
| Proportion receiving no antimicrobial | 78.6% | 35.9% | Vignettes survey[3] |

## Prescription Cost Calculations

Antibiotic regimens commonly used for varicella patients were oral clindamycin, cefuroxime, amoxicillin and cephalexin.[4] The cost of each drug regimen was calculated as the average cost of oral suspension for a younger child (25kg) and capsule/tablet for an older child (50kg) for an average of 8.5 days (7-10 days) using goodrx.com. The average cost of treatment with oral antibiotics was calculated as $92.54 and the average cost of antiviral regimens (acyclovir or valacyclovir, 5 days regimens) was similarly calculated as $108.32.

Note: The total prescription cost depends on the average cost per regimen that we calculated. Though we used commonly prescribed drugs and costs from goodrx, using different drugs and/or costs will impact the results. To be conservative, we used only oral drug costs (instead of any intravenous) and used average costs to get as unbiased an estimate as possible. Since the prescribed dose depends on weight, we calculated average costs for f a 25 kg younger child and 50 kg older child. Complications are more likely to occur with increasing age and will need more expensive treatment. Our model does not account for age dependent costs apart from the calculation of dose based on body weight.

### Table C. Average antiviral treatment cost

| **A** | **B** | **C** | **D** | **E** | **F** |
| --- | --- | --- | --- | --- | --- |
| **Drug name** | **Patient Type** | **Total mg needed for treatment regimen**  **[C]** | **Avg retail price for GoodRx prescription**  **[D]** | **Total mgs in GoodRx prescription for that price**  **[E]** | **Total Cost to Treat Varicella Infection**  **(F=C*D/E)** |
| ^1^Acyclovir  tablet | Adolescent | 16,000 mg | $44.44 | 24,000mg  (400mg x 60 count) | **$29.63** |
| ^2^Acyclovir oral suspension | Child | 10,000 mg | $264.33 | 12,000 mg  (300ml x 200mg/5ml) | **$220.28** |
| ^3^Valacyclovir tablet | Adolescent | 15,000 mg | $122.26 | 15,000 mg  (500mg x 30 count) | **$122.26** |
| ^4^Valacyclovir tablet crushed | Child | 7,500mg | $122.26 | 15,000 mg  (500mg x 30 count) | **$61.13** |
| **Average Retail Pharmacy Costs for Antiviral prescriptions** | | | | | **$108.32*** |

All sources accessed January 7, 2021:

^1^https://www.goodrx.com/acyclovir?dosage=400mg&form=tablet&label_override=acyclovir&quantity=60&sort_type=popularity; (800mg x 4 times/day x 5 days= 16,000 mg (flat mg/kg since dose not to exceed 800mg/dose)

^2^https://www.goodrx.com/acyclovir?dosage=200mg-5ml&form=oral-suspension&label_override=acyclovir&quantity=210&sort_type=popularity; 20mk/kg x 4 times/day x 5 days x 25Kg = 10,000 mg

^3^https://www.goodrx.com/valacyclovir?dosage=500mg&form=tablet&label_override=valacyclovir&quantity=30&sort_type=popularity; 1000 mg x 3 times/day x 5 days= 15,000 mg (note: dose not to exceed 1000 mg /dose) Tablet

^4^Rate used from above. 20mk/kg x 3 times/day x 5 days x 25Kg = 7,500mg.

*Final number due to rounding.

### Table D. Average antibiotic treatment cost

| **Drug name** | **Patient Type** | Average mg per kg per day  [C] | Average weight of child (kg)  [D] | Average days of therapy  [E] | Total mgs in treatment period  [F=C x D x E] | **Avg retail price for GoodRx prescription**  [G] | **Total mgs in GoodRx prescription for that price**  [H] | **Total Cost to Treat Varicella Infection**  [I=F*G/H] |
| --- | --- | --- | --- | --- | --- | --- | --- | --- |
| ^1^Clindamycin (generic) capsule | Adolescent | 25 | 50 | 8.5 | 10,625 | $98.53 | 12,000  (300mg x 40 count) | **$87.24** |
| ^2^Clindamycin (generic)  Oral Suspension | Child | 25 | 25 | 8.5 | 5,313 | $232.16 | 6,000  (100ml x 75mg/5ml x 4 bottles) | **$205.56** |
| ^3^Cefuroxime (generic)  capsule | Adolescent | 30 | 50 | 8.5 | 12,750 | $84.22 | 15,000  (250mg x 60 count) | **$71.59** |
| ^4^Cefuroxime (Branded Ceftin)  Oral Suspension | Child | 30 | 25 | 8.5 | 6,375 | $160.50 | 7,500  (100ml x125mg/5ml x  3 bottles) | **$136.43** |
| ^5^Amoxicillin (generic)  Chewable Tablet | Adolescent | 85 | 50 | 8.5 | 36,125 | $87.14 | 40,000  (250mg x 160 count) | **$78.70** |
| ^6^Amoxicillin (generic)  Oral Suspension | Child | 85 | 25 | 8.5 | 18,063 | $43.06 | 24,000  (100ml x 400mg/5ml x 3 bottles) | **$32.41** |
| ^7^Cephalexin (generic)  capsule | Adolescent | 40 | 50 | 8.5 | 17,000 | $30.47 | 20,000  (250mg x 40 count) | **$25.90** |
| ^8^Cephalexin (generic)  Oral Suspension | Child | 40 | 25 | 8.5 | 8,500 | $120.64 | 10,000  (100ml x 125mg/5ml x 4 bottles) | **$102.54** |
| **Average Retail Pharmacy Costs for Antibiotic prescriptions** | | | | | | | | **$92.54** |

All sources accessed January 7, 2021:

^1^https://www.goodrx.com/clindamycin?dosage=300mg&form=capsule&label_override=clindamycin&quantity=40&sort_type=popularity

^2^https://www.goodrx.com/clindamycin?dosage=100ml-of-75mg-5ml&form=bottle-of-oral-solution&label_override=clindamycin&quantity=4&sort_type=popularity

^3^https://www.goodrx.com/cefuroxime-axetil?dosage=250mg&form=tablet&label_override=cefuroxime+axetil&quantity=60&sort_type=popularity

^4^https://www.goodrx.com/cefuroxime-axetil?dosage=100ml-of-125mg-5ml&form=bottle-of-oral-
suspension&label_override=Ceftin&quantity=3&sort_type=popularity; goodrx did not provide an average cost, but all pharmacies showed a range from $437-$475 for 3 bottles

^5^https://www.goodrx.com/amoxicillin?dosage=250mg&form=chewable-tablet&label_override=amoxicillin&quantity=160&sort_type=popularity

^6^https://www.goodrx.com/amoxicillin?dosage=100ml-of-400mg-5ml&form=bottle-of-oral-suspension&label_override=amoxicillin&quantity=3&sort_type=popularity

^7^https://www.goodrx.com/cephalexin?dosage=500mg&form=capsule&label_override=cephalexin&quantity=40&sort_type=popularity

^8^https://www.goodrx.com/cephalexin?dosage=100ml-of-250mg-5ml&form=bottle-of-oral-suspension&label_override=cephalexin&quantity=4&sort_type=popularity

## Vignettes used for Calculations

### Table E. Vignettes used for analysis^[[1]](#footnote-1)^

| **Vignette** | **Varicella vaccination status** | **Diagnosis** | **Primary Treatment Recommendation** |
| --- | --- | --- | --- |
| **1** | **Unvaccinated** healthy 3.5-year-old with no known exposure to varicella | Varicella with no complications | Supportive care |
| **2** | Healthy 5-year-old with **1-dose vaccination**, exposed 14 days prior | Varicella with no complications | Supportive care |
| **3** | **Unvaccinated** healthy 7-year-old not exposed to varicella | Varicella with **Complication** (*S. aureus* infection of lesions) | **Antibiotics**  +/- Supportive care |
| **4** | **Unvaccinated** 3-year-old with history of asthma and on prednisone, exposed to shingles 7 days prior | Varicella with no complications | **Antivirals**  +/- Supportive care |
| **5** | **Unvaccinated** healthy 10-month-old, exposed to varicella 12 days prior | Varicella with no complications | Supportive care |
| **6** | **Unvaccinated** healthy 15-year-old with a personal belief exemption, exposed to varicella 10 days prior | Varicella with no complications | **Antivirals**  +/- Supportive care |
| **7** | Healthy 6-year-old, with **1-dose vaccination** experiencing respiratory sign/symptoms and no known exposure to varicella | Varicella with  **Complication** (pneumonia) | **Antibiotics**  +**Hospitalization**  +/- Supportive care |
| **8** | **Unvaccinated** healthy 14-year-old with a religious belief exemption and no known exposure to varicella. | Varicella with no complications | Supportive care |

## Modeling results

### Table F. Base case scenario: Annual varicella prescriptions and costs for total, varicella cases with and without complications

| **BASE CASE**  **(Per year)** | **Unvaccinated** | | **1-Dose Vaccination** | | **2-Dose Vaccination** | | Total | |
| --- | --- | --- | --- | --- | --- | --- | --- | --- |
| All Varicella Cases | | | | | | | | |
| **Total Varicella Cases** | **396,633** | **100%** | **14,406** | **100%** | **140,395** | **100%** | **551,434** | **100%** |
| - Only antiviral (AV) Prescribed | 63,045 | 15.9% | 2,321 | 16.1% | 22,798 | 16.2% | 88,164 | 16.0% |
| - Only antibiotic (AB) Prescribed | 29,823 | 7.5% | 848 | 5.9% | 7,986 | 5.7% | 38,657 | 7.0% |
| - Both AV & AB Prescribed | 3,943 | 1.0% | 107 | 0.7% | 883 | 0.6% | 4,934 | 0.9% |
| - Neither AB nor AV | 299,821 | 75.6% | 11,130 | 77.3% | 108,729 | 77.4% | 419,680 | 76.1% |
| **Total antiviral + antibiotic prescriptions** | **100,755** | **100.0%** | **3,383** | **100.0%** | **32,550** | **100.0%** | **136,687** | **100.0%** |
| - Total antivirals prescriptions | 66,989^#^ | 66.5% | 2,428 | 71.8% | 23,681 | 72.8% | 93,098^#^ | 68.1% |
| - Total antibiotic prescriptions | 33,766 | 33.5% | 955 | 28.2% | 8,869 | 27.2% | 43,590 | 31.9% |
|  |  |  |  |  |  |  |  |  |
| Cases prescribed either antiviral or antibiotic | 96,812 | 24.4% | 3,276 | 22.7% | 31,667 | 22.6% | 131,754 | 23.9% |
| Variella Cases with no complications | | | | | | | | |
| **Total Cases with no complications** | **369,625** | **100%** | **14,029** | **100%** | **137,490** | **100%** | **521,143** | **100%** |
| - Only antiviral (AV) Prescribed | 59,251 | 16.0% | 2,268 | 16.2% | 22,342 | 16.3% | 83,861 | 16.1% |
| - Only antibiotic (AB) Prescribed | 18,622 | 5.0% | 692 | 4.9% | 6,785 | 4.9% | 26,100 | 5.0% |
| - Both AV & AB Prescribed | 1,628 | 0.4% | 71 | 0.5% | 628 | 0.5% | 2,327 | 0.4% |
| - Neither AB nor AV | 290,124 | 78.5% | 10,997 | 78.4% | 107,735 | 78.4% | 408,856 | 78.5% |
| **Total antiviral + antibiotic prescriptions** | **81,129** | **100.0%** | **3,102** | **100.0%** | **30,383** | **100.0%** | **114,614** | **81,129** |
| - Total antivirals prescriptions | 60,879 | 75.0% | 2,339 | 75.4% | 22,970 | 75.6% | 86,188 | 75.2% |
| - Total antibiotic prescriptions | 20,250 | 25.0% | 763 | 24.6% | 7,413 | 24.4% | 28,426 | 24.8% |
|  |  |  |  |  |  |  |  |  |
| Cases prescribed either antiviral or antibiotic | 79,501 | 21.5% | 3,031 | 21.6% | 29,755 | 83.8% | 112,288 | 21.5% |
| Varicella Cases with complications | | | | | | | | |
| **Total Cases with complications** | **27,007** | **100%** | **378** | **100%** | **2,906** | **100%** | **30,291** | **100%** |
| - Antiviral (AV) Prescribed | 3,794 | 14.0% | 53 | 14.0% | 456 | 15.7% | 4,303 | 14.2% |
| - Antibiotic (AB) Prescribed | 11,200 | 41.5% | 156 | 41.3% | 1,201 | 41.3% | 12,557 | 41.5% |
| - Both AV & AB Prescribed | 2,316 | 8.6% | 36 | 9.5% | 255 | 8.8% | 2,607 | 8.6% |
| - Neither AB nor AV | 9,697 | 35.9% | 133 | 35.2% | 994 | 34.2% | 10,824 | 35.7% |
| **Total antiviral + antibiotic prescriptions** | **19,626** | **100.0%** | **281** | **100.0%** | **2,167** | **100.0%** | **22,074** | **100.0%** |
| - Total antivirals prescriptions | 6,110 | 31.1% | 89 | 31.7% | 711 | 32.8% | 6,910 | 31.3% |
| - Total antibiotic prescriptions | 13,516 | 68.9% | 192 | 68.3% | 1,456 | 67.2% | 15,164 | 68.7% |
|  |  |  |  |  |  |  |  |  |
| Cases prescribed either antiviral or antibiotic | 17,310 | 64.1% | 245 | 64.8% | 1,912 | 65.8% | 19,467 | 64.3% |

**Base case: Current vaccination scenario in the US with unvaccinated: 3.8%; 1 dose: 2.4%; 2 doses: 93.8%.

### Table G. Annual prescription cost for base case scenarios

| BASE CASE SCENARIO | **Unvaccinated** | **1-Dose Vaccination** | **2-Dose Vaccination** | **Total** |
| --- | --- | --- | --- | --- |
| **TOTAL** | | | | |
| Antiviral prescription cost | $7,256,248 | $263,001 | $2,565,126 | **$10,084,375** |
| Antibiotic prescription cost | $3,124,706 | $88,376 | $820,737 | **$4,033,819** |
| Total prescription costs | $10,380,954 | $351,377 | $3,385,863 | **$14,118,194** |
| Row % | 73.5% | 2.5% | 24% | **100%** |
| Average cost per case | $26.17 | $24.39 | $24.12 | **$25.60** |
| CASES WITH NO COMPLICATIONS | | | | |
| Antiviral prescription cost | $6,594,413 | $253,360 | $2,488,110 | **$9,335,884** |
| Antibiotic prescription cost | $1,873,935 | $70,608 | $685,999 | **$2,630,542** |
| Total prescription costs | $8,468,348 | $323,969 | $3,174,109 | **$11,966,426** |
| Average cost per case | $22.91 | $23.09 | $23.09 | **$22.96** |
| CASES WITH COMPLICATIONS | | | | |
| Antiviral prescription cost | $661,835 | $9,640 | $77,016 | **$748,491** |
| Antibiotic prescription cost | $1,250,771 | $17,768 | $134,738 | **$1,403,277** |
| Total prescription costs | $1,912,606 | $27,408 | $211,754 | **$2,151,768** |
| Average cost per case | $70.82 | $72.59 | **$72.87** | **$71.04** |

###

### Table H. Annual varicella prescriptions for scenarios with varying vaccination coverage (Base case, 0%, 20%, 80% vaccination coverage scenarios)

| **Base Case** | | | | | | | | |  |
| --- | --- | --- | --- | --- | --- | --- | --- | --- | --- |
| Base case scenario | **Unvaccinated**  **N, row%** | | **1-Dose Vaccination**  **N, row%** | | **2-Dose Vaccination**  **N, row%** | | Total **N, row%** | | |
| Varicella cases | 396,633 | 71.9% | 14,406 | 2.6% | 140,395 | 25.5% | **551,434** | **100%** | |
| Cases prescribed either antiviral or antibiotic | 96,812 | 73.5% | 3,276 | 2.5% | 31,667 | 24.0% | **131,754** | **100%** | |
| Total antivirals prescriptions | 66,989 | 72.0% | 2,428 | 2.6% | 23,681 | 25.4% | **93,098^#^** | **100.0%** | |
| Total antibiotic prescriptions | 33,766 | 77.5% | 955 | 2.2% | 8,869 | 20.3% | **43,590** | **100.0%** | |
| Total antiviral + antibiotic prescriptions | 100,754 | 73.7% | 3,383 | 2.5% | 32,550 | 23.8% | **136,688^#^** | **100.0%** | |
| **0% Vaccinated** | | | | | | | | | |
| **0% Vaccinated** | **Unvaccinated** | | **1-Dose Vaccination** | | **2-Dose Vaccination** | | Total | | |
| Varicella cases | 10,437,699 | 100.0% | - | - | - | - | **10,437,699** | 100.0% | |
| Cases prescribed either antiviral or antibiotic | 2,547,672 | 100.0% | - | - | - | - | **2,547,672** | 100.0% | |
| Total antivirals prescriptions | 1,762,865 | 100.0% | - | - | - | - | **1,762,865** | 100.0% | |
| Total antibiotic prescriptions | 888,581 | 100.0% | - | - | - | - | **888,581** | 100.0% | |
| Total antiviral + antibiotic prescriptions | 2,651,446 | 100.0% | - | - | - | - | **2,651,446** | 100.0% | |
| **20% Vaccinated** | | | | | | | | | |
| **20% Vaccinated** | **Unvaccinated** | | **1-Dose Vaccination** | | **2-Dose Vaccination** | | **Total** | | |
| Varicella cases | 8,350,159 | 99.6% | 2,995 | 0.0% | 29,188 | 0.3% | **8,382,343** | 100.0% | |
| Cases prescribed either antiviral or antibiotic | 2,038,138 | 99.6% | 681 | 0.0% | 6,584 | 0.3% | **2,045,402** | 100.0% | |
| Total antivirals prescriptions | 1,410,292 | 99.6% | 505 | 0.0% | 4,923 | 0.3% | **1,415,720** | 100.0% | |
| Total antibiotic prescriptions | 710,865 | 99.7% | 199 | 0.0% | 1,844 | 0.3% | **712,907** | 100.0% | |
| Total antiviral + antibiotic prescriptions | 2,121,157 | 99.6% | 703 | 0.0% | 6,767 | 0.3% | **2,128,627** | 100.0% | |
| **80% Vaccinated** | | | | | | | | | |
| **80% Vaccinated** | **Unvaccinated** | | **1-Dose Vaccination** | | **2-Dose Vaccination** | | **Total** | | |
| Varicella cases | 2,087,540 | 94.2% | 11,980 | 0.5% | 116,753 | 5.3% | **2,216,273** | 100.0% | |
| Cases prescribed antiviral or antibiotic | 509,534 | 94.6% | 2,724 | 0.5% | 26,334 | 4.9% | **538,593** | 100.0% | |
| Total antivirals prescriptions | 352,573 | 94.2% | 2,019 | 0.5% | 19,693 | 5.3% | **374,285** | 100.0% | |
| Total antibiotic prescriptions | 177,716 | 95.6% | 794 | 0.4% | 7,376 | 4.0% | **185,886** | 100.0% | |
| Total antiviral + antibiotic prescriptions | 530,289 | 94.7% | 2,813 | 0.5% | 27,069 | 4.8% | **560,171** | 100.0% | |

^# Rounding difference of 1^

### Table I. Annual varicella prescription costs for scenarios with varying vaccination coverage (Base case, 0%, 20%, 80% vaccination coverage scenarios)

| Base Case | | | | |
| --- | --- | --- | --- | --- |
| **Base case scenario** | **Unvaccinated** | **1-Dose Vaccination** | **2-Dose Vaccination** | Total |
| Antiviral RX Costs | $7,256,248 | $263,001 | $2,565,126 | $10,084,375 |
| Antibiotic RX Costs | $3,124,706 | $88,376 | $820,737 | $4,033,819 |
| Total Prescription Costs | $10,380,954 | $351,377 | $3,385,863 | $14,118,194 |
| Mean Prescription Cost Per Case | $26.17 | $24.39 | $24.12 | $25.60 |
| 0% Vaccinated | | | | |
| **0% Vaccinated** | **Unvaccinated** | **1-Dose Vaccination** | **2-Dose Vaccination** | Total |
| Antiviral RX Costs | $190,953,537 | $ - | $ - | $190,953,537 |
| Antibiotic RX Costs | $82,229,286 | $ - | $ - | $82,229,286 |
| Total Prescription Costs | $273,182,823 | $ - | $ - | $273,182,823 |
| Mean Prescription Cost Per Case | $26.17 | $ - | $ - | $26.17 |
| 20% Vaccinated | | | | |
| **20% Vaccinated** | **Unvaccinated** | **1-Dose Vaccination** | **2-Dose Vaccination** | Total |
| Antiviral RX Costs | $152,762,829 | $54,702 | $533,259 | $153,350,790 |
| Antibiotic RX Costs | $65,783,447 | $18,415 | $170,644 | $65,972,414 |
| Total Prescription Costs | $218,546,277 | $73,117 | $703,903 | $219,323,204 |
| Mean Prescription Cost Per Case | $26.17 | $24.41 | $24.12 | $26.16 |
| 80% Vaccinated | | | | |
| **80% Vaccinated** | **Unvaccinated** | **1-Dose Vaccination** | **2-Dose Vaccination** | Total |
| Antiviral RX Costs | $38,190,707 | $218,698 | $2,133,146 | $40,542,551 |
| Antibiotic RX Costs | $16,445,839 | $73,477 | $682,575 | $17,201,890 |
| Total Prescription Costs | $54,636,546 | $292,175 | $2,815,721 | $57,744,442 |
| Mean Prescription Cost Per Case | $26.17 | $24.39 | $24.12 | $26.05 |

*** Total Prescription cost is the sum of antiviral and antibiotic prescription costs used for varicella patients.

**Base case: Current vaccination scenario in USA with unvaccinated: 3.8%; 1 dose: 2.4%; 2 doses: 93.8%.

***Average costs of antibiotic and antiviral was estimated to be $92.54 and $108.32 respectively

### Table J. Data for Figure 2: Annual prescriptions for varicella cases with no complications

| **Figure 2:** Annual prescriptions for varicella cases with no complications | **100% Unvaccinated** | **80% Unvaccinated** | **20% Unvaccinated** | **Base Case** | **Decrease in prescriptions in base case compared to 0% vac. scenario** | **% Decrease in base case compared to 0% vac. scenario** |
| --- | --- | --- | --- | --- | --- | --- |
| Annual Antiviral prescriptions | 1,602,079 | 1,286,925 | 341,463 | 86,188 | 1,515,891 | 94.62% |
| Annual Antibiotic prescriptions | 532,896 | 428,016 | 113,378 | 28,426 | 504,470 | 94.67% |
| Total | 2,134,975 | 1,714,941 | 454,841 | 114,614 | 2,020,361 | 94.63% |

### Table K. Data for Figure 3: Annual prescriptions for varicella cases with complications

| **Figure 3: Annual prescriptions for varicella cases with complications** | **0% Unvaccinated** | **80% Unvaccinated** | **20% Unvaccinated** | **Base Case** | **Decrease in prescriptions in base case compared to 0% vac. scenario** | **% Decrease in base case compared to 0% vac. scenario** |
| --- | --- | --- | --- | --- | --- | --- |
| Annual Antiviral prescriptions | 160,786 | 128,795 | 32,822 | 6,910 | 153,877 | 95.70% |
| Annual Antibiotic prescriptions | 355,686 | 284,891 | 72,508 | 15,164 | 340,521 | 95.74% |
| Total | 516,472 | 413,686 | 105,330 | 22,074 | 494,398 | 95.73% |

### Table L. Data for Figure 4: Annual varicella prescription costs

| Annual Costs ($) | **0% vaccinated** | **20% vaccinated** | **80% vaccinated** | **Base Case** | **Cost saving in base case compared to 0% vaccination scenario** | **% reduction compared to 0% vaccination scenario** |
| --- | --- | --- | --- | --- | --- | --- |
| Annual Antiviral costs | $190,953,537 | $153,350,790 | $40,542,551 | $10,084,375 | $180,869,161 | 94.72% |
| Annual Antibiotic costs | $82,229,286 | $65,972,414 | $17,201,890 | $4,033,819 | $78,195,467 | 95.09% |
| Total Prescription costs | $273,182,823 | $219,323,204 | $57,744,442 | $14,118,194 | $259,064,629 | 94.83% |

## References

1. Mellerson, J.L., et al., *Vaccination Coverage for Selected Vaccines and Exemption Rates Among Children in Kindergarten - United States, 2017-18 School Year.* MMWR Morb Mortal Wkly Rep, 2018. **67**(40): p. 1115-1122.

2. Kuter, B., et al., *Ten year follow-up of healthy children who received one or two injections of varicella vaccine.* Pediatr Infect Dis J, 2004. **23**(2): p. 132-7.

3. Fergie, J., et al., *1387. Current practices in the diagnosis and treatment of varicella infections in the United States.* Open Forum Infectious Diseases, 2020. **7**(Supplement_1): p. S703-S704.

4. Mészner, Z., et al., *Burden of varicella in Central and Eastern Europe: findings from a systematic literature review.* Expert Review of Vaccines, 2019. **18**(3): p. 281-293.

1. Fergie J, Pawaskar MD, Veeranki P, et al. 1387. Current practices in the diagnosis and treatment of varicella infections in the United States. Open Forum Infectious Diseases. 2020;7(Supplement_1):S703-S4. doi: 10.1093/ofid/ofaa439.1569 [↑](#footnote-ref-1)
