# Supplementary material for: Impact of universal varicella vaccination on the use and cost of antibiotics and antivirals for varicella management in the United States
Source: PLoS One. 2022 Jun 10;17(6):e0269916. doi: 10.1371/journal.pone.0269916 (PMC9187103; doi:10.1371/journal.pone.0269916)
Supplement: Attachment — Submitted filename: Response to reviewers AMR 2 5.4.22.docx [file pone.0269916.s002.docx]

May 4^th^ , 2022

To,

Georges M.G.M. Verjans, MSc, PhD
Academic Editor,
PLOS ONE

Reference: PONE-D-21-40227 Response to reviewers

Dear Dr. Verjans,

On behalf of my co-authors, we are pleased to resubmit our manuscript entitled “**Impact of universal varicella vaccination on the use and cost of antibiotics and antivirals for varicella management in the United States**” for publication review in *Plos One*.

We appreciate the valuable comments and suggestions on our manuscript provided by the editor and reviewers. We have carefully studied each comment, provided point-by-point responses, and revised our manuscript accordingly. The line numbers provided correspond to the line numbers in the tracked changes version of the manuscript. We hope that these revisions meet your approval.

In addition, the corporate address of our company has changed, and the funding statement should be revised as “*This study was sponsored by Merck Sharp & Dohme LLC, a subsidiary of Merck & Co., Inc., Rahway, NJ, USA (MSD). The funder had no role in study design, data collection and analysis, decision to publish, or preparation of the manuscript. M. Pawaskar and S. Samant, are employees of MSD and own stocks in Merck & Co., Inc., Rahway, NJ, USA. P. Veeranki, and C. Harley, are employees of PRECISIONheor, which received financial support from MSD, for the execution of this research. Though they received no payment for their work on this study, J. H. Conway reports grants and personal fees from Sanofi Pasteur, Pfizer, Merck, GSK, and Centers for Disease Control outside of the submitted work while J. Fergie reports personal fees from MSD, outside the submitted work.”*

Part of this research was presented as an oral presentation at the European Society for Paediatric Infectious Diseases (ESPID) Virtual Meeting, May 24-29, 2020*.* However, the slides were not peer reviewed and were not published. The data was updated since then.

We look forward to hearing from you.

Best regards,

On behalf of the co-authors,

Manjiri Pawaskar, PhD

Merck Sharp & Dohme LLC, a subsidiary of Merck & Co., Inc., Rahway, NJ, USA

email: manjiri.pawaskar@merck.com

____________________________________________________________________________________________

Additional Editor Comments (if provided):

Dear Dr Pawaskar and colleagues,

Your manuscript has been reviewed by two experts in the field. Both reviewers and I agree that your manuscript is well written and of interest to the field. Minor revisions are requested to improve the manuscript, please do so per reviewers' suggestions.

Best regards,

Georges MGM Verjans
[www.herpeslab.nl](https://nam12.safelinks.protection.outlook.com/?url=http%3A%2F%2Fwww.herpeslab.nl%2F&data=04%7C01%7Ccarolyn.harley%40precisionformedicine.com%7Ca3e527aeecd84606aaf408da0c2a03a9%7Cb71ff3f628164ca8a9b938f820f91ad1%7C0%7C0%7C637835671847793653%7CUnknown%7CTWFpbGZsb3d8eyJWIjoiMC4wLjAwMDAiLCJQIjoiV2luMzIiLCJBTiI6Ik1haWwiLCJXVCI6Mn0%3D%7C3000&sdata=Hxh0MndJtLLxQF1gqL6jV9dJxNHAnfDLpgpCYScui3E%3D&reserved=0)
Dept. Viroscience
Rotterdam
The Netherlands


[Note: HTML markup is below. Please do not edit.]

Reviewers' comments:

Reviewer's Responses to Questions

**Comments to the Author**

1. Is the manuscript technically sound, and do the data support the conclusions?

The manuscript must describe a technically sound piece of scientific research with data that supports the conclusions. Experiments must have been conducted rigorously, with appropriate controls, replication, and sample sizes. The conclusions must be drawn appropriately based on the data presented.

Reviewer #1: Yes

Reviewer #2: Yes

2. Has the statistical analysis been performed appropriately and rigorously?

Reviewer #1: Yes

Reviewer #2: Yes

3. Have the authors made all data underlying the findings in their manuscript fully available?

The [PLOS Data policy](https://nam12.safelinks.protection.outlook.com/?url=http%3A%2F%2Fwww.plosone.org%2Fstatic%2Fpolicies.action%23sharing&data=04%7C01%7Ccarolyn.harley%40precisionformedicine.com%7Ca3e527aeecd84606aaf408da0c2a03a9%7Cb71ff3f628164ca8a9b938f820f91ad1%7C0%7C0%7C637835671847793653%7CUnknown%7CTWFpbGZsb3d8eyJWIjoiMC4wLjAwMDAiLCJQIjoiV2luMzIiLCJBTiI6Ik1haWwiLCJXVCI6Mn0%3D%7C3000&sdata=3rAwQ45rkSEwxFoJQ534dengaepnGEuODN2SGx%2BF4gQ%3D&reserved=0) requires authors to make all data underlying the findings described in their manuscript fully available without restriction, with rare exception (please refer to the Data Availability Statement in the manuscript PDF file). The data should be provided as part of the manuscript or its supporting information or deposited to a public repository. For example, in addition to summary statistics, the data points behind means, medians and variance measures should be available. If there are restrictions on publicly sharing data—e.g., participant privacy or use of data from a third party—those must be specified.

Reviewer #1: Yes

Reviewer #2: Yes

4. Is the manuscript presented in an intelligible fashion and written in standard English?

PLOS ONE does not copyedit accepted manuscripts, so the language in submitted articles must be clear, correct, and unambiguous. Any typographical or grammatical errors should be corrected at revision, so please note any specific errors here.

Reviewer #1: Yes

Reviewer #2: Yes

5. Review Comments to the Author

Please use the space provided to explain your answers to the questions above. You may also include additional comments for the author, including concerns about dual publication, research ethics, or publication ethics. (Please upload your review as an attachment if it exceeds 20,000 characters)

**Reviewer #1**: This manuscript presents a version of a cost-benefit analysis for varicella vaccination in the United States in 2017. The authors show the expected cost reductions associated with universal vaccination in the USA. The goal of the report appears to be an opportunity to convince other countries to authorize universal varicella vaccination. A few comments are listed below.

1. Results, Varicella cases, lines 124-8 and Table 1. This table contains numbers that are not widely known among vaccine experts. The authors state that 93.8% of children in the USA were vaccinated against varicella in 2017. Yet the authors estimate that 396,633 cases of wild-type varicella (chickenpox) occurred in the remaining unvaccinated population of children. Please write more in the text about how the authors arrived at the number of 396,633. Furthermore, the authors estimate that 140,395 cases of break-through varicella occurred in the fully vaccinated children. Please write more in the text about how the authors arrived at the number of 140,395.

Response:

Thank you for your comment. We have revised the manuscript as follows: *“Under current vaccination coverage rates, a total of 551,434 cases of varicella were estimated to occur annually among US children < 18 years of age with 396,633 varicella cases among unvaccinated children, 14,406 cases among those vaccinated with a single dose and 140,395 cases among those vaccinated with 2 doses of varicella vaccine (See Table A in S1 Appendix for details).).* Lines # 132-135

In addition*,* we have added stepwise explanation along with appropriate references and footnotes explaining how we calculated 396,633 annual varicella cases among unvaccinated children, 14,406 cases among those vaccinated with a single dose and 140,395 varicella cases among those with 2 dose vaccinations in the revised Table A in S1 Appendix.

### Table A. Model inputs for varicella cases by vaccination status for a cross-section of 2017 cohort

| Row | Definition | Unvaccinated | 1-Dose Vaccinated | 2-Dose Vaccinated | Total | Source |
| --- | --- | --- | --- | --- | --- | --- |
| #1 | Proportion of children <18 years of age in 2017 by vaccination status | 3.8% | 2.4% | 93.8% | 100% | Mellerson 2018[1],  See footnote ^a^ |
| #2 | Population distribution of children <18 years of age in 2017 by vaccination status | 2,796,155 | 1,765,992 | 69,020,868 | 73,583,015 | U.S. Census Bureau, 2017;  See footnote ^b^ |
| #3 | Average annual incidence rate by vaccination status | 14.0% | 0.8% | 0.2% | NA | Kuter 2004[2] |
| #4 | Number of varicella cases in 2017 by vaccination status | 396,633 | 14,406 | 140,395 | 551,434 | See footnote ^c^ |
| #5 | Average annual rate of varicella complications | 6.7% | 2.5% | 2.0% | NA | Derived from Kuter 2004[2] |
| #6 | Annual number of varicella cases with complications | 27,007 | 378 | 2,906 | 30,291 | See footnote ^d^ |
| #7 | Annual number of varicella cases with no complications | 369,625 | 14,029 | 137,490 | 521,143 | See footnote ^e^ |

* Numbers in this table are rounded up.

Calculations

^a) From Table 1 of Mellerson at al 2018 for the 2017-2018 school year, the median 2-dose varicella vaccination coverage for the country was 93.8% ; while that for 1 dose varicella vaccination was 96.2%.[1] This was used to calculate proportion of children of <18 year olds in 2017 vaccinated with a single dose of varicella vaccine (96.2% - 93.8% = 2.4%); and the proportion who were unvaccinated (remaining = 100% - 93.8% - 2.4% = 3.8%) in Row#1^

^b) Population per US Census of individuals <18 years of age in 2017=73,583,015. This number was multiplied by 3.8%, 2.4% and 93.8% respectively to get the population by vaccination status in 2017 among <18 year olds in the US in Row#2^

^c) To calculate the number of varicella cases in 2017 by vaccination status in Row#4, the populations in Row #2 for unvaccinated, 1 dose and 2 dose groups were multiplied by their respective average annual incidence rates in Row#3. Summing the number of varicella cases across the 3 groups = 551,434 total varicella cases in 2017 among children <18 years in 2017^

^d) Derived from row #4 and #5^ ^for each vaccination group and then summed up.^

^e) To calculate the number of varicella cases with no complications, subtract Row #6 from Row#4^

2. Add new section near the top of the Discussion about varicella herd immunity. If the number of cases of varicella in Table 1 are correct, the USA does not appear to have achieved herd immunity for varicella, even with an immunization rate of 93.8%. This is a striking conclusion, especially when we are at the end of the COVID-19 epidemic. In the USA, about 65% of the population has received complete COVID-19 vaccination. Can the authors state whether they think varicella or COVID-19 is more contagious? If they are roughly equally contagious, based on this report, the USA will never achieve herd immunity against COVID-19 by immunization, since we will never achieve greater than 92% COVID-19 vaccination. After the COVID-19 epidemic, there appears to be ever greater resistance to vaccination in the USA. Please discuss the above points in a new paragraph in the Discussion about what is meant by herd immunity and whether we have achieved herd immunity to varicella in the USA in 2017?

Response:

Thank you for your comment.

- A total number of 551,434 varicella cases estimated were among children <18 years old in 2017 (total cohort, n=73,583,015) so the number of estimated varicella cases is still small 0.75%. There is also published data that show herd immunity due to varicella vaccination in the US. The manuscript text is edited in response to this comment to explain more about varicella herd immunity with appropriate references. The total number of cases reported This text was added to the discussion section as part of the limitations. (Lines # 248- 268)

*“This is a cross-section predictive model consists of only pediatric population and not an epidemiological model with entire US population. It does not account for the indirect benefits among the unvaccinated (including adults) through herd immunity effects. Hence, this model cannot provide any insights on the herd immunity with varicella vaccination. However, there is strong real world evidence supporting herd immunity due to varicella vaccination in the US with significant reductions reported in varicella incidence and varicella related hospitalizations even among the unvaccinated. ([3-7]). Although herd immunity for varicella is achieved in the US, VZV can also be transmitted to unvaccinated children from adults with herpes zoster; however, it is less contagious than that from patients with varicella. [8] A household study reported that 16% of susceptible children <15 years old exposed to herpes zoster developed varicella. [9] Some clinical parameters used in our model such as the annual incidence of varicella and incidence of cases with complications after 1 and 2 doses were derived from randomized control trials, which did not account for herd immunity effects. Hence, our model estimated a larger number of varicella cases annually (551,434 cases i.e. 0.75% of children under 18 years of age in 2017) than current estimates reported by the CDC (350,000 cases annually) [10] . Hence, our model may have underestimated the benefits and cost-savings related to vaccination and provided more conservative estimates of benefits of vaccination.”*

- It is difficult to draw meaningful comparisons between vaccination and COVID-19 and the effects of vaccination on herd immunity in the US.
  - Unlike COVID-19 which affects both children and adults, the incidence of varicella is much higher among children with >90% of infections in a temperate country like the US, occurring before adolescence. [11]
  - Varicella vaccination is targeted towards children and 2 doses of commonly used varicella vaccines are highly efficacious (98% efficacy for 2-doses) with no waning observed over 14 years follow up period [4]. This is unlike COVID-19 vaccine with vaccine effectiveness that may wane, requiring a booster within 1 year.
  - Though Reproduction number Ro can give an idea of how contagious the disease is, lockdowns may lead to underestimation of Ro in COVID-19. The Ro for delta variant of COVID-19 is estimated to be 5.08[12] which is said to be as transmissible as Chicken pox, though estimates vary.[13-15]

3. Abstract. Suggest that one sentence about herd immunity be added into the Abstract. Was there herd immunity in the USA in 2017 against wild type varicella? Presumably herd immunity would never be achievable with an 80% immunization rate?

Response:

- We have added “*and did not account for herd immunity benefits*” to the abstract (Lines # 33) and the manuscript methods section (Lines # 96-98)
- In addition, we are addressed the question of herd immunity in more detail in the discussion section as follows and explain why our model underestimates the benefits and cost-savings of vaccination compared to no-vaccination scenario (Lines # 248- 268)

*“This is a cross-section predictive model consists of only pediatric population and not an epidemiological model with entire US population. It does not account for the indirect benefits among the unvaccinated (including adults) through herd immunity effects. Hence, this model cannot provide any insights on the herd immunity with varicella vaccination. However, there is strong real world evidence supporting herd immunity due to varicella vaccination in the US with significant reductions reported in varicella incidence and varicella related hospitalizations even among the unvaccinated. ([3-7]). Although herd immunity for varicella is achieved in the US, VZV can also be transmitted to unvaccinated children from adults with herpes zoster; however, it is less contagious than that from patients with varicella. [8] A household study reported that 16% of susceptible children <15 years old exposed to herpes zoster developed varicella. [9] Some clinical parameters used in our model such as the annual incidence of varicella and incidence of cases with complications after 1 and 2 doses were derived from randomized control trials, which did not account for herd immunity effects. Hence, our model estimated a larger number of varicella cases annually (551,434 cases i.e. 0.75% of children under 18 years of age in 2017) than current estimates reported by the CDC (350,000 cases annually) [10] . Hence, our model may have underestimated the benefits and cost-savings related to vaccination and provided more conservative estimates of benefits of vaccination.”*

Reviewer #2: The aim of this study was to estimate the use of antivirals and antibiotics for treating varicella in children. The impact of vaccination was modeled after the experience in the U.S., where Varivax coverage is high. The use of antimicrobial therapy was estimated from a prescriber survey of 8 clinical vignettes. The parameters of the model were taken from published sources, although the actual reported varicella cases from the CDC were not used. This model was also used to predict the cost savings from reduced prescriptions in countries where vaccine coverage was absent, intermediate, or high. The findings presented here are interesting and compelling, although they should be taken as estimates only. These results could be used for future policy decisions on implementing varicella vaccine guidelines.

- Line 127: correct typo, remove “were”.
- Line 149: correct typo “d”.
- Line 225: correct typo “nay”.

Response:

Thank you for pointing out these typos. We have updated the manuscript accordingly.

- Line 127: corrected typo, removed “were”. (Revised Lines#137)
- Line 149: corrected typo “d”. (Revised Line#161)
- Line 225: corrected typo “nay”. (Revised Line #239)

Supplemental Table 6 contains the key estimates of the model. However, it is difficult to find the “Total Varicella Cases” and the “Total Cases with no complications” and further down the “Total Cases with complications”. These rows are shaded gray, and the font is bold. Why are these values not the top rows of the entire table? Why are they embedded in other rows that show the number of cases treated with antivirals or antibiotics? Every other row is a subset of these two key estimates, which form the base cases from the model.

Response:

Thank you. We agree that the ‘Total varicella cases”, “Total cases with no complications” and “Total cases with complications” are most important and hence are shown in Table 1 of the main manuscript (shown below for your reference, Rows #2,#3 and #4)

Table 1. Estimated annual varicella cases, antibiotic prescriptions and antiviral prescriptions in the US population under 18 years old by vaccination status, base case

|  | **Unvaccinated** | **1-Dose**  **(partial)** | | **2-Dose**  **(full)** | **Total** |
| --- | --- | --- | --- | --- | --- |
| Population | 2,796,155  (3.8%) | 1,765,992  (2.4%) | | 69,020,868 (93.8%) | 73,583,015  (100%) |
| Varicella cases  (% of population)  (Row %) | 396,633  (14.2%)  (71.9%) | 14,406  (0.8%)  (2.6%) | | 140,395  (0.2%)  (25.5%) | 551,434  (0.7%)  (100%) |
| Cases with no complications (% of Cases) | 369,625  (93.2%) | 14,029  (97.4%) | | 137,490  (97.9%) | 521,143  (94.5%) |
| Cases with complications  (% of Cases) | 27,007  (6.8%) | 378  (2.6%) | | 2,906  (2.1%) | 30,291  (5.5%) |
| Cases with antiviral or antibiotic prescription  (% of cases) | 96,812  (24.4%) | 3,276  (22.7%) | | 31,667  (22.6%) | 131,754  (23.9%) |
| Antiviral prescription only  (% of cases)  (row %) | 66,988  (16.9%)  (72.0%) | 2,428  (16.9%)  (2.6%) | | 23,681  (16.9%)  (25.4%) | 93,098  (16.9%)  (100%) |
| Antibiotic prescription only  (% of cases)  (row %) | 33,766  (8.5%)  (77.5%) | 955  (6.6%)  (2.2%) | 8,869  (6.3%)  (20.3%) | | 43,590  (7.9%)  (100%) |
| Total prescriptions  (Row %) | 100,755  (73.8%) | 3,383  (2.5%) | 32,550  (23.8%) | | 136,688  (100%) |

Base case scenario. Results were extrapolated to the 2017 population of 73.5 million US children (0-18 years) with vaccination coverage as follows: unvaccinated: 3.8%; 1 dose: 2.4%; 2 doses: 93.8%. See supplement for details.

The Table F in Supplement Appendix 1 looks at Annual varicella prescriptions and costs for total, varicella cases with and without complications and focus on annual varicella prescriptions and costs for base case scenario. The total cases and number of cases with no complications and complications are provided to allow a better understanding of how the prescription use added up to 100%. However, we have updated the Table F in Appendix S1 as advised, by moving the totals to the top and using highlight/bold font.

| **BASE CASE**  **(Per year)** | **Unvaccinated** | | **1-Dose Vaccination** | | **2-Dose Vaccination** | | Total | |
| --- | --- | --- | --- | --- | --- | --- | --- | --- |
| All Varicella Cases | | | | | | | | |
| **Total Varicella Cases** | **396,633** | **100%** | **14,406** | **100%** | **140,395** | **100%** | **551,434** | **100%** |
| - Only antiviral (AV) Prescribed | 63,045 | 15.9% | 2,321 | 16.1% | 22,798 | 16.2% | 88,164 | 16.0% |
| - Only antibiotic (AB) Prescribed | 29,823 | 7.5% | 848 | 5.9% | 7,986 | 5.7% | 38,657 | 7.0% |
| - Both AV & AB Prescribed | 3,943 | 1.0% | 107 | 0.7% | 883 | 0.6% | 4,934 | 0.9% |
| - Neither AB nor AV | 299,821 | 75.6% | 11,130 | 77.3% | 108,729 | 77.4% | 419,680 | 76.1% |
| **Total antiviral + antibiotic prescriptions** | **100,755** | **100.0%** | **3,383** | **100.0%** | **32,550** | **100.0%** | **136,687** | **100.0%** |
| - Total antivirals prescriptions | 66,989^#^ | 66.5% | 2,428 | 71.8% | 23,681 | 72.8% | 93,098^#^ | 68.1% |
| - Total antibiotic prescriptions | 33,766 | 33.5% | 955 | 28.2% | 8,869 | 27.2% | 43,590 | 31.9% |
|  |  |  |  |  |  |  |  |  |
| Cases prescribed either antiviral or antibiotic | 96,812 | 24.4% | 3,276 | 22.7% | 31,667 | 22.6% | 131,754 | 23.9% |
| Variella Cases with no complications | | | | | | | | |
| **Total Cases with no complications** | **369,625** | **100%** | **14,029** | **100%** | **137,490** | **100%** | **521,143** | **100%** |
| - Only antiviral (AV) Prescribed | 59,251 | 16.0% | 2,268 | 16.2% | 22,342 | 16.3% | 83,861 | 16.1% |
| - Only antibiotic (AB) Prescribed | 18,622 | 5.0% | 692 | 4.9% | 6,785 | 4.9% | 26,100 | 5.0% |
| - Both AV & AB Prescribed | 1,628 | 0.4% | 71 | 0.5% | 628 | 0.5% | 2,327 | 0.4% |
| - Neither AB nor AV | 290,124 | 78.5% | 10,997 | 78.4% | 107,735 | 78.4% | 408,856 | 78.5% |
| **Total antiviral + antibiotic prescriptions** | **81,129** | **100.0%** | **3,102** | **100.0%** | **30,383** | **100.0%** | **114,614** | **81,129** |
| - Total antivirals prescriptions | 60,879 | 75.0% | 2,339 | 75.4% | 22,970 | 75.6% | 86,188 | 75.2% |
| - Total antibiotic prescriptions | 20,250 | 25.0% | 763 | 24.6% | 7,413 | 24.4% | 28,426 | 24.8% |
|  |  |  |  |  |  |  |  |  |
| Cases prescribed either antiviral or antibiotic | 79,501 | 21.5% | 3,031 | 21.6% | 29,755 | 83.8% | 112,288 | 21.5% |
| Varicella Cases with complications | | | | | | | | |
| **Total Cases with complications** | **27,007** | **100%** | **378** | **100%** | **2,906** | **100%** | **30,291** | **100%** |
| - Antiviral (AV) Prescribed | 3,794 | 14.0% | 53 | 14.0% | 456 | 15.7% | 4,303 | 14.2% |
| - Antibiotic (AB) Prescribed | 11,200 | 41.5% | 156 | 41.3% | 1,201 | 41.3% | 12,557 | 41.5% |
| - Both AV & AB Prescribed | 2,316 | 8.6% | 36 | 9.5% | 255 | 8.8% | 2,607 | 8.6% |
| - Neither AB nor AV | 9,697 | 35.9% | 133 | 35.2% | 994 | 34.2% | 10,824 | 35.7% |
| **Total antiviral + antibiotic prescriptions** | **19,626** | **100.0%** | **281** | **100.0%** | **2,167** | **100.0%** | **22,074** | **100.0%** |
| - Total antivirals prescriptions | 6,110 | 31.1% | 89 | 31.7% | 711 | 32.8% | 6,910 | 31.3% |
| - Total antibiotic prescriptions | 13,516 | 68.9% | 192 | 68.3% | 1,456 | 67.2% | 15,164 | 68.7% |
|  |  |  |  |  |  |  |  |  |
| Cases prescribed either antiviral or antibiotic | 17,310 | 64.1% | 245 | 64.8% | 1,912 | 65.8% | 19,467 | 64.3% |

**Base case: Current vaccination scenario in the US with unvaccinated: 3.8%; 1 dose: 2.4%; 2 doses: 93.8%.

In the Discussion, the point is raised that the estimate of total varicella cases may be high compared to the CDC estimates. How much higher? The CDC estimate should be included here, and the justification not to use that value should be explained. In fact, herd immunity will have a strong effect on the actual number of varicella cases in the U.S., because outbreaks of chicken pox are becoming rare. More often, varicella arises in unvaccinated children (or infants <1) who are exposed to adults with zoster. A more nuanced discussion of this situation could be added to the manuscript.

Thank you. Although CDC reported total annual varicella cases were 350,000 we couldn’t use this data in the model. As per model structure we need data on varicella cases by vaccination status (no vaccination, 1-dose and 2-dose vaccination) and by complications (with and without complications). The latest epidemiological data on these subgroups is not available in the literature since the implementation of universal vaccination program in the US, hence we used the incidence data reported in the Kuter et al study to calculate these estimates. We have updated the manuscript to include the CDC estimate and rationale for different numbers and added the following text to Lines # 248- 268

*” This is a cross-section predictive model consists of only pediatric population and not an epidemiological model with entire US population.* *It does not account for the indirect benefits among the unvaccinated (including adults) through herd immunity effects. Hence, this model cannot provide any insights on the herd immunity with varicella vaccination. However, there is strong real world evidence supporting herd immunity due to varicella vaccination in the US with significant reductions reported in varicella incidence and varicella related hospitalizations even among the unvaccinated. ([3-7]). Although herd immunity for varicella is achieved in the US, VZV can also be transmitted to unvaccinated children from adults with herpes zoster; however, it is less contagious than that from patients with varicella. [8] A household study reported that 16% of susceptible children <15 years old exposed to herpes zoster developed varicella. [9] Some clinical parameters used in our model such as the annual incidence of varicella and incidence of cases with complications after 1 and 2 doses were derived from randomized control trials, which did not account for herd immunity effects. Hence, our model estimated a larger number of varicella cases annually (551,434 cases i.e. 0.75% of children under 18 years of age in 2017) than current estimates reported by the CDC (350,000 cases annually) [10] . Hence, our model may have underestimated the benefits and cost-savings related to vaccination and provided more conservative estimates of benefits of vaccination.”*

6. PLOS authors have the option to publish the peer review history of their article ([what does this mean?](https://nam12.safelinks.protection.outlook.com/?url=https%3A%2F%2Fjournals.plos.org%2Fplosone%2Fs%2Feditorial-and-peer-review-process%23loc-peer-review-history&data=04%7C01%7Ccarolyn.harley%40precisionformedicine.com%7Ca3e527aeecd84606aaf408da0c2a03a9%7Cb71ff3f628164ca8a9b938f820f91ad1%7C0%7C0%7C637835671847793653%7CUnknown%7CTWFpbGZsb3d8eyJWIjoiMC4wLjAwMDAiLCJQIjoiV2luMzIiLCJBTiI6Ik1haWwiLCJXVCI6Mn0%3D%7C3000&sdata=kC%2BFoX%2BpWcAJJFYOI9q9bg8fQb4AhCmWl5Y23ZVonNY%3D&reserved=0)). If published, this will include your full peer review and any attached files.


If you choose “no”, your identity will remain anonymous but your review may still be made public.


**Do you want your identity to be public for this peer review?** For information about this choice, including consent withdrawal, please see our [Privacy Policy](https://nam12.safelinks.protection.outlook.com/?url=https%3A%2F%2Fwww.plos.org%2Fprivacy-policy&data=04%7C01%7Ccarolyn.harley%40precisionformedicine.com%7Ca3e527aeecd84606aaf408da0c2a03a9%7Cb71ff3f628164ca8a9b938f820f91ad1%7C0%7C0%7C637835671847793653%7CUnknown%7CTWFpbGZsb3d8eyJWIjoiMC4wLjAwMDAiLCJQIjoiV2luMzIiLCJBTiI6Ik1haWwiLCJXVCI6Mn0%3D%7C3000&sdata=2rlAxZVj5oN72bjfUdEx1eUCDTBg7JMVHkCh5WFpTpU%3D&reserved=0).

Reviewer #1: No

Reviewer #2: No

[NOTE: If reviewer comments were submitted as an attachment file, they will be attached to this email and accessible via the submission site. Please log into your account, locate the manuscript record, and check for the action link "View Attachments". If this link does not appear, there are no attachment files.]

While revising your submission, please upload your figure files to the Preflight Analysis and Conversion Engine (PACE) digital diagnostic tool, [https://pacev2.apexcovantage.com/](https://nam12.safelinks.protection.outlook.com/?url=https%3A%2F%2Fpacev2.apexcovantage.com%2F&data=04%7C01%7Ccarolyn.harley%40precisionformedicine.com%7Ca3e527aeecd84606aaf408da0c2a03a9%7Cb71ff3f628164ca8a9b938f820f91ad1%7C0%7C0%7C637835671847793653%7CUnknown%7CTWFpbGZsb3d8eyJWIjoiMC4wLjAwMDAiLCJQIjoiV2luMzIiLCJBTiI6Ik1haWwiLCJXVCI6Mn0%3D%7C3000&sdata=zLxo3mBTwfOu%2Fjw%2FRiyuuMKaLbv%2FkoFwebDCFVbUVRk%3D&reserved=0). PACE helps ensure that figures meet PLOS requirements. To use PACE, you must first register as a user. Registration is free. Then, login and navigate to the UPLOAD tab, where you will find detailed instructions on how to use the tool. If you encounter any issues or have any questions when using PACE, please email PLOS at [figures@plos.org](mailto:figures@plos.org). Please note that Supporting Information files do not need this step.

References:

- 1.	Mellerson, J.L., et al., *Vaccination Coverage for Selected Vaccines and Exemption Rates Among Children in Kindergarten - United States, 2017-18 School Year.* MMWR Morb Mortal Wkly Rep, 2018. **67**(40): p. 1115-1122.
- 2.	Kuter, B., et al., *Ten year follow-up of healthy children who received one or two injections of varicella vaccine.* Pediatr Infect Dis J, 2004. **23**(2): p. 132-7.
- 3.	Varela, F.H., L.A. Pinto, and M.C. Scotta, *Global impact of varicella vaccination programs.* Human vaccines & immunotherapeutics, 2019. **15**(3): p. 645-657.
- 4.	Baxter, R., et al., *Long-term effectiveness of varicella vaccine: a 14-Year, prospective cohort study.* Pediatrics, 2013. **131**(5): p. e1389-96.
- 5.	Baxter, R., et al., *Impact of vaccination on the epidemiology of varicella: 1995–2009.* Pediatrics, 2014. **134**(1): p. 24-30.
- 6.	Leung, J. and R. Harpaz, *Impact of the maturing varicella vaccination program on varicella and related outcomes in the United States: 1994–2012.* Journal of the Pediatric Infectious Diseases Society, 2016. **5**(4): p. 395-402.
- 7.	Bialek, S.R., et al., *Impact of a routine two-dose varicella vaccination program on varicella epidemiology.* Pediatrics, 2013. **132**(5): p. e1134-40.
- 8.	Schmid, D.S. and A.O. Jumaan, *Impact of varicella vaccine on varicella-zoster virus dynamics.* Clinical microbiology reviews, 2010. **23**(1): p. 202-217.
- 9.	Seiler, H., *A study of herpes zoster particularly in its relationship to chickenpox.* Epidemiology & Infection, 1949. **47**(3): p. 253-262.
- 10.	CDC. *Chickenpox Vaccine Saves Lives and Prevents Serious Illness Infographic*. 2021; Available from: <https://www.cdc.gov/chickenpox/vaccine-infographic.html>.
- 11.	Organization, W.H., *Varicella and herpes zoster vaccines: WHO position paper, June 2014.* Weekly Epidemiological Record= Relevé épidémiologique hebdomadaire, 2014. **89**(25): p. 265-287.
- 12.	Liu, Y. and J. Rocklöv, *The reproductive number of the Delta variant of SARS-CoV-2 is far higher compared to the ancestral SARS-CoV-2 virus.* Journal of travel medicine, 2021.
- 13.	Lee, T., et al., *Estimating the basic reproductive number of varicella in South Korea incorporating social contact patterns and seroprevalence.* Human Vaccines & Immunotherapeutics, 2021. **17**(8): p. 2488-2493.
- 14.	Nardone, A., et al., *The comparative sero-epidemiology of varicella zoster virus in 11 countries in the European region.* Vaccine, 2007. **25**(45): p. 7866-7872.
- 15.	Abutaleb, Y., C.Y. Johnson, and J. Achenbach, *The war has changed’: Internal CDC document urges new messaging, warns delta infections likely more severe.* Washington Post, 2021.
